# Neural correlates of verbal creativity: differences in resting-state functional connectivity associated with expertise in creative writing

**DOI:** 10.3389/fnhum.2014.00516

**Published:** 2014-07-15

**Authors:** Martin Lotze, Katharina Erhard, Nicola Neumann, Simon B. Eickhoff, Robert Langner

**Affiliations:** ^1^Functional Imaging Unit, Center for Diagnostic Radiology and Neuroradiology, University of GreifswaldGreifswald, Germany; ^2^Institute of Clinical Neuroscience and Medical Psychology, Heinrich Heine University DüsseldorfDüsseldorf, Germany; ^3^Institute of Neuroscience and Medicine, Research Centre JülichJülich, Germany

**Keywords:** creativity, expertise, resting-state-fMRI, functional connectivity, temporal pole, interhemispheric connectivity, basal ganglia, brain

## Abstract

Neural characteristics of verbal creativity as assessed by word generation tasks have been recently identified, but differences in resting-state functional connectivity (rFC) between experts and non-experts in creative writing have not been reported yet. Previous electroencephalography (EEG) coherence measures during rest demonstrated a decreased cooperation between brain areas in association with creative thinking ability. Here, we used resting-state functional magnetic resonance imaging to compare 20 experts in creative writing and 23 age-matched non-experts with respect to rFC strengths within a brain network previously found to be associated with creative writing. Decreased rFC for experts was found between areas 44 of both hemispheres. Increased rFC for experts was observed between right hemispheric caudate and intraparietal sulcus. Correlation analysis of verbal creativity indices (VCIs) with rFC values in the expert group revealed predominantly negative associations, particularly of rFC between left area 44 and left temporal pole. Overall, our data support previous findings of reduced connectivity between interhemispheric areas and increased right-hemispheric connectivity during rest in highly verbally creative individuals.

## Introduction

Creativity is considered as the ability to produce original and unexpected work, which is appropriate for a given goal (Stein, 1953). Recent reviews (Dietrich, 2007; Abraham, 2013) emphasized the need for cognitive models that define different aspects of creativity and specify the underlying cognitive processes. In a preliminary framework to distinguish between branches of creativity, Abraham (2013) grouped a problem-solving domain and an expression domain. In that framework, creative expression denotes the ability to express oneself in a unique manner. Within the expression domain, there are subgroups depending on the nature of the task (verbal, art, music, etc.). Referring to this framework, the present study represents a between-group approach, in which groups differ with regard to their ability to write creative texts (verbal expression domain).

Two previous studies have investigated the neural correlates of creative story writing. In a positron-emission-tomography study, Bechtereva et al. (2004) found left parieto-temporal regions (BA 39, 40) active during a difficult story generation condition in comparison to an easier one as well as conditions that controlled for syntactic and memory related aspects of the task. The authors concluded that these areas are required to provide the necessary flexibility for creative thinking. In contrast, Howard-Jones et al. (2005) using fMRI found right prefrontal areas as well as the anterior cingulate cortex associated with creative versus uncreative story generation. These activations were connected to episodic retrieval, monitoring, and higher cognitive control. Neither of the two studies investigating story generation, however, involved actual writing in the scanner.

When using a text continuation task, we recently demonstrated that creative writing involved bilateral hippocampi, temporal poles (BA 38) and the cingulate cortex (CC; Shah et al., 2013). These areas have been associated with episodic memory retrieval, free-associative and spontaneous cognition and semantic integration. In addition, there were correlations of the verbal creativity index (VCI; Schoppe, 1975) with activations in the left inferior frontal gyrus (BA 44), the left middle frontal gyrus (BA 9/46) and the left temporal pole (BA 38). In a recent study (Erhard et al., 2014), we compared functional activation during creative writing in groups of expert and non-expert writers using the same paradigm. Experts showed increased left-hemispheric activation in the caudate nucleus and superior medial prefrontal cortex.

Apart from task-related activation sites, studies on the interaction between brain areas in specific networks are informative, especially in creativity research (Jung et al., 2013). Functional connectivity is defined as the statistical association among two or more anatomically distinct time-series (Friston et al., 1993) and can *inter alia* be assessed with electroencephalography (EEG) coherence measures or fMRI resting state functional connectivity. Generally, creative achievement has been connected to decreased cortical arousal, as demonstrated by an increased EEG alpha power (Martindale and Hines, 1975; Fink and Benedek, 2012) and reduced scores on “latent inhibition”, the capacity to screen from conscious awareness stimuli previously experienced as irrelevant (Carson et al., 2003). EEG coherence measures determined during rest have indicated less cooperation between brain areas in more creative individuals. This decoupling of brain areas has been found equally distributed over the right and left hemispheres, showing also significant interhemispheric decoupling (Jausovec and Jausovec, 2000). fMRI resting-state functional connectivity (rFC) studies revealed that higher creativity scores were associated with an increased rFC between the medial prefrontal cortex (mPFC) and the posterior cingulate cortex (Takeuchi et al., 2012), which was linked to a stronger interaction within the default mode network (Raichle et al., 2001). Wei et al. (2014) found a positive correlation of rFC between the left mPFC and the left middle temporal gyrus with creativity scores that was interpreted as representing another hub associated with the default mode network. Increased rFC between the mPFC and the posterior cingulate cortex, however, was not confirmed. Taken together, the two existing studies on rFC and creativity were not consistent, but both identified connections associated with the default mode network.

In contrast to the above-mentioned studies, in the present study we investigated rFC in a group of experts in creative writing and compared it to non-experts. rFC was not only correlated to creativity scores assessed by normative creativity tests, but also to the rating of the actual texts written inside the scanner. Seeds for FC analysis were selected from activation maxima calculated during a text continuation task performed by participants included in two previous studies (Shah et al., 2013; Erhard et al., 2014). These areas were the pars opercularis of the inferior frontal gyrus (area 44), the CC, the superior temporal gyrus (STG), the hippocampus, the caudate nucleus (caudate), and the intraparietal sulcus (IPS). According to the findings of EEG-resting state studies, we hypothesized a decreased interhemispheric and left hemispheric FC and a more right hemispheric FC in the expert writers.

## Methods

### Participants

We investigated 43 native German participants. Twenty expert students of Creative Writing and Culture Journalism from the only two universities in Germany that offer academic courses in creative writing: the Universities of Hildesheim and Leipzig (8 females and 12 males; mean age: 25.2, standard deviation (±) 2.7; mean semester: 7.1 ± 3.9). These students can be considered as well-selected and domain-specific talented people, because the selection criteria for the programs are extremely competitive and only 6% percent of applications are accepted. Twenty-three students from the University of Greifswald (non-experts in creative writing; 11 female and 12 male; mean age: 24.0 ± 1.9) formed the control group. For the non-expert group, we investigated 22 students of medicine, four students of the humanities (psychology, history, english, philosophy), one student from the faculty of law, and one student from the faculty of business. All participants were right-handed (as assessed by the Edinburgh Handedness Inventory; Oldfield, 1971) and reported no history of neurological or psychiatric disorders. Written informed consent was obtained from all participants before entering the study, which was approved by the Ethics Committee of the Medical Faculty of the University of Greifswald.

### Expertise measures

All participants were asked about their experience and practice of creative writing. Experts reported writing experience of 11.7 ± 4.8 years on average, including their studies of creative writing, whereas the non-experts claimed an average of 3.1 ± 5.2 years. Weekly writing practice during the last three months before scanning amounted to 21.0 ± 10.2 h for the expert and 0.5 ± 0.8 h for the non-expert group (*t*_(19)_ = 9.0; *p* < 0.001). Likewise, experts had more years of experience (*t*_(46)_ = 5.80; *p* < 0.001). Adapting a method commonly used in music research, we calculated an individual “practice index” (PI) by multiplication of creative writing experience with weekly writing practice [(semester + years of writing practice) × practice of writing per week].

### Task

All participants continued a text of two different literary texts (text A written by Ror Wolf; text B written by Durs Grünbein) over a time of 2 min and 20 s, respectively. In accordance with the CAT (Amabile, 1996), all produced texts were typewritten and sent in a randomized order to four independent judges, who were generally familiar with the domain (two professors and two lecturers from the department of Creative Writing and Culture Journalism at the University of Hildesheim). All judges rated the creativity of each text on a 10-cm-long visual analog scale (VAS; from 0: not creative at all, to 10: extremely creative). The creative writing performance in the scanner (creative writing ranking; CR) was calculated for every participant using the mean value of both texts A and B of the “creativity” rating from all judges.

The verbal creativity test (Schoppe, 1975) yielding a summary VCI, consisted of nine subtests analyzing the participants’ verbal fluency and verbal production skills, whereas some subtests were also including aspects of flexibility and originality. We evaluated these verbal creativity tests according to its standardized instructions.

### Definition of the seed regions related to creative writing

The regions of interest (“seeds”) for the present investigation had previously been identified by fMRI observed during a text continuation task (Shah et al., 2013; Erhard et al., 2014). The main effects for both groups were calculated and thresholded at *p* < 0.05 (false discovery rate (FDR)-corrected for multiple comparisons across the whole brain). The following seeds showed significance and were therefore tested in the present study: left posterior area 44 (MNI coordinates: −57, 6, 27), right posterior area 44 (54, 6, 33), medial cingulate cortex (−9, 12, 42), left IPS (−36, −39, 48), right IPS (42, −33, 45), left hippocampus (−27, −9, −24), left temporal pole (−48, 9, −18), left posterior superior temporal sulcus (STS) (−54, −42, 6), left (−9, 3, 15), and right (15, 21, 15) caudate nucleus. The peak activation foci of each cluster were taken as centers of spheres with 5 mm radius to define the volumes of interest for the present analysis.

### Magnetic resonance imaging

Data were acquired at a 3T Siemens Magnetom Verio (Siemens, Erlangen, Germany) with a 32-channel headcoil. Two-dimensional echo-planar images (EPI) were acquired with repetition time TR = 2000 ms, echo time TE = 30 ms, flip angle = 90°, field of view = 192 × 192 mm^2^. Each volume consisted of 34 slices with a voxel size of 3 × 3 × 3 mm^3^ with a 1-mm gap between them. The first two volumes of each run were discarded to allow for T1 equilibration effects.

We used baseline scans interspersed in an experimental block design alternating task-related activation and rest. Overall, each of six different activation conditions was presented twice to each participant. Experimental blocks between baseline periods used for resting-state analysis were five blocks of 60 s duration (experimental conditions: reading, copying, silent speech, brainstorming, correcting) and one block of 140 s duration (creative writing). Participants were presented instructions on a scanner-adapted desk. A double mirror affixed on the headcoil enabled the view on the in-scanner desk with the instruction sheets, the text material, the writing sheet, and the fixation cross. During rest, participants were instructed to stop thinking about the task and fixate a fixation cross (eyes-open baseline). Rest periods had a total duration of 20 s (10 volumes). The first three scans of each baseline period were not used in order to reduce BOLD effects from the activation period. The procedure for using baseline scans from block design fMRI experiments was adapted for our purpose from Fair et al. (2007). For rFC analysis, 90 volumes for each participant were used (5 × 8 volumes before and between blocks, plus five volumes at the end of each scanning session). Details on fluctuations during baseline have been described more recently by Garrett et al. (2010). In total, 90 baseline scans of resting state data from two consecutive measurement runs were available for analysis.

### Resting-state connectivity analysis

Data were jointly preprocessed using SPM8 (Wellcome Department of Cognitive Neuroscience, London, UK). Images were first corrected for head movement by affine registration using a two-pass procedure by which images were initially realigned to the first image and subsequently to the mean of the realigned images. Each participant’s mean image was then spatially normalized to the Montreal Neurological Institute (MNI) single-subject template brain using the “unified segmentation” approach (Ashburner and Friston, 2005), and the ensuing deformation was applied to the individual EPI volumes. Hereby, volumes were resampled at 1.5 × 1.5 × 1.5 × mm^3^ voxel size. Images were then smoothed by a 5-mm full-width at half-maximum Gaussian kernel to increase the signal-to-noise ratio and compensate for remaining differences in individual anatomy.

rFC measures can be influenced by several confounds such as head movements and physiological processes (e.g., fluctuations due to cardiac and respiratory cycles; cf. Fox et al. (2009)). In order to reduce spurious correlations, variance explained by the following nuisance variables was removed from each voxel’s BOLD signal time series (for a detailed evaluation of this procedure see Satterthwaite et al. (2013)): (i) the six motion parameters derived from the image realignment; (ii) the first derivatives of the six motion parameters; (iii) mean tissue-class specific signal intensity per time point (Cieslik et al., 2013). All nuisance variables entered the regression model as first- and second-order terms, resulting in a total of 30 nuisance regressors. After confound removal, data were band-pass filtered preserving frequencies between 0.01 and 0.08 Hz, as meaningful resting-state correlations will predominantly be found in these frequencies given that the BOLD response acts as a low-pass filter (Greicius et al., 2003).

The time course of each seed region’s BOLD signal was then extracted for each participant as the first eigenvariate of activity in all gray-matter voxels located within the respective seed. For each participant, the time-series data of each seed region were correlated with each other, and the resulting Pearson correlation coefficients were transformed into Fisher’s *Z* scores. Subsequently, the influence of age and sex was partialled out of both the resting-state correlations and the covariates of interest. Main effects of rFC (across the entire sample) were tested by one-sample *t*-tests, applying a significance threshold of *p* < 0.05 (adjusted for multiple comparisons by FDR correction). Median rFC in experts and non-experts was compared via a non-parametric approach using 10,000 realizations of the null hypothesis (group-label exchangeability) in a Monte-Carlo simulation to create an empirical null distribution of group differences (posterior-probability significance threshold: *p* > 0.95, uncorrected). Additionally, we applied effect-size criteria: first, differences were only considered potentially relevant if the rFC score in either group (or both) corresponded at least to a small effect (i.e., *r* ≥ 0.10). Second, the between-group difference in rFC itself needed to correspond to a large effect (i.e., Cohen’s *d* ≥ 0.80) to be considered relevant here. Finally, creativity-related changes in interregional coupling were examined by rank-correlating participants’ Fisher-*Z*-transformed rFC values with creativity scores across both the entire sample and the expert subgroup alone. The results of these Spearman correlation analyses were regarded significant if they passed a threshold of *p* < 0.05 (uncorrected). Again, we applied an effect-size criterion: accordingly, correlations were regarded as relevant if they were of at least medium size according to Cohen’s effect-size categorization (i.e., *r* ≥ 0.24).

## Results

### Creativity scores

Mean VCI (Schoppe, 1975) was 116.5 ± 9.9 for expert writers and 107.1 ± 8.8 for non-experts (*t*_(46)_ = 3.42; *p* < 0.01). Creative performance of experts (creativity rating; CR) was commonly judged higher than those of non-experts (*t*_(46)_ = 3.36, *p* < 0.01). We observed a positive correlation between creative performance in the scanner and individual verbal creativity scores (CR and VCI: *r* = 0.38, *p* < 0.01). Expert writers had much more experience in writing creative texts (PI) than non-experts (*t*_(19)_ = 6.24; *p* < 0.001), and this correlated positively with performance (PI and CR: *r* = 0.46, *p* < 0.01) and individual verbal creativity (PI and VCI: *r* = 0.43, *p* < 0.01).

### Basic resting-state functional connectivity (rFC)

**Figure 1:**
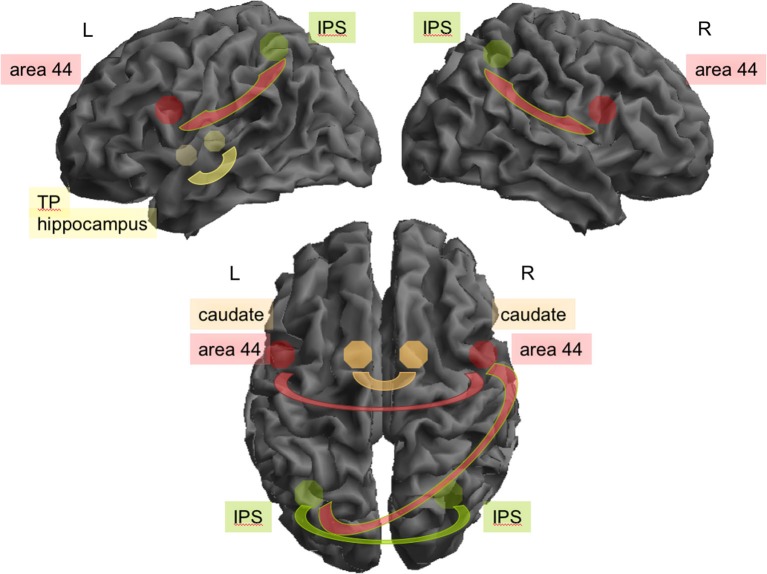
**Connectivity between seeds found to be relevant during rest for all participants**. Top: In the left (L) hemisphere area 44 (red) and the intraparietal sulcus (green; IPS; *z* = 5.14) and hippocampus (yellow) and temporal pole (yellow, TP; *z* = 3.77) were highly interconnected. On the right (R) hemisphere the IPS and area 44 (*z* = 4.87). Interhemispheric connectivity (bottom) was relevant for bilateral IPS (*z* = 5.37), left IPS and right area 44 (*z* = 4.91), between both caudate (orange; *z* = 3.19) and between both areas 44 (*z* = 3.09).

Across both groups, there was significant positive rFC between the following regions: bilateral IPS, area 44, and caudate nucleus, respectively, were all highly interconnected between hemispheres (IPS: *z* = 5.37; areas 44: *z* = 3.09; caudate nuclei: *z* = 3.19). IPS was additionally coupled to ipsilateral areas 44 on both hemispheres (right: *z* = 4.87, left: *z* = 5.14), and left IPS was coupled with right area 44 (*z* = 4.91). The left hippocampus and the left temporal pole also showed high rFC (*z* = 3.77; see Figure 1). In addition, left area 44 was significantly interconnected with MCC (*z* = 2.82).

### Resting-state FC differences between experts and non-experts

**Figure 2:**
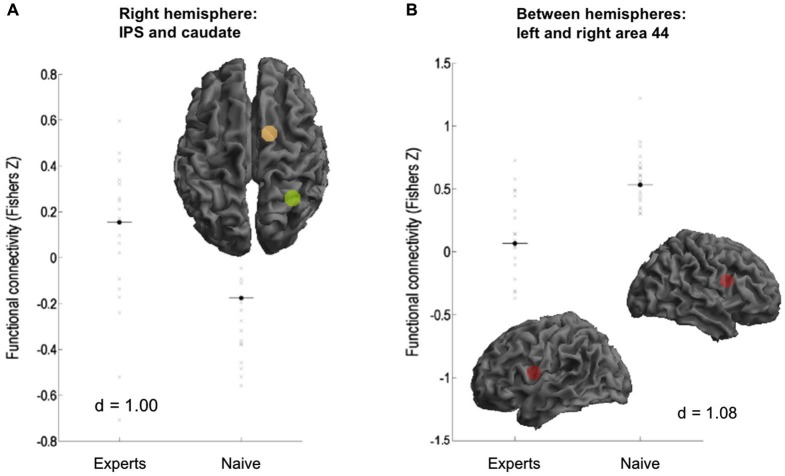
**Functional connectivity differences between the subject groups. (A)** Experts showed increased FC between right intraparietal sulcus (IPS) and caudate. **(B)** Naive showed increased connectivity between area 44 of both hemispheres.

Experts showed significantly increased rFC between right IPS and right caudate nucleus (*p* > 0.99, *d* = 1.00; see Figure 2A). Furthermore, experts showed reduced rFC between hemispheres in bilateral area 44 (*p* > 0.99, *d* = 1.08; see Figure 2B), between right area 44 and left IPS (*p* = 0.99, *d* = 1.12), as well as between right area 44 and left caudate nucleus (*p* = 0.97, *d* = 0.83).

### Correlations between creativity scores and rFC across all participants

We observed positive associations of the creativity ratings of the texts (CR) with rFC between left IPS and right caudate (*r* = 0.31, *p* = 0.041). Negative correlations of CR with rFC were found between left area 44 and right IPS (*r* = −0.36; *p* = 0.018) as well as rFC between left area 44 and left aSTG (*r* = −0.35; *p* = 0.022). Furthermore, we observed several negative correlations between the VCI and FC, specifically interhemispheric rFC between bilateral area 44 (*r* = −0.43; *p* = 0.004) as well as rFC between left temporal pole and left caudate (*r* = −0.37; *p* = 0.015), left IPS and left hippocampus (*r* = −0.34; *p* = 0.03), and right IPS and MCC (*r* = −0.31; *p* = 0.041).

### Correlations between creativity scores and rFC in experts

**Figure 3:**
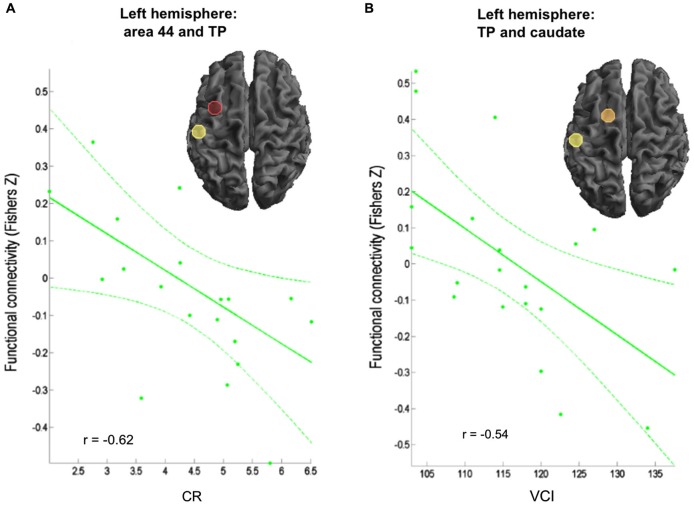
**Correlation of behavioral data and FC in the expert group. (A)** Negative correlation between verbal creativity rating (Amabile, 1996) and connectivity between left area 44 and left temporal pole (TP). **(B)** Negative correlation between creativity index (CI; Schoppe, 1975) and connectivity between left TP and caudate.

In experts, rFC between left area 44 and left temporal pole correlated negatively with CR (*r* = −0.62; *p* = 0.004; Figure 3A), while a positive correlation of CR was observed with rFC between right area 44 and left posterior STS (*r* = 0.55; *p* = 0.013) as well as rFC between left IPS and right caudate (*r* = 0.45; *p* = 0.0498). Furthermore, rFC between left temporal pole and left caudate was negatively associated with VCI (*r* = −0.54; *p* = 0.014; Figure 3B). Conversely, rFC between left IPS and right caudate were positively correlated with VCI (*r* = 0.44; *p* = 0.0496) in experts. Comparisons of the correlations between creativity scores and rFC in experts and non-experts yielded no significant results.

## Discussion

We here compared rFC between experts and non-experts in the field of creative writing. Experts showed considerable higher creativity scores in the verbal creativity tests and the creativity ratings of their written texts. These differences in performance might therefore well be associated with differential connectivity during rest. For investigating rFC, we did not investigate the default mode network as recently done by others (Wei et al., 2014), but used seed regions based on our previous studies with the same story generation task (Shah et al., 2013; Erhard et al., 2014). Across both groups, interhemispheric rFC during rest was high between the inferior parietal sulci, the caudate nuclei, and the areas 44. Additionally, the IPS was significantly connected to the ipsilateral area 44 on both hemispheres, as well as the left hippocampus to the left temporal pole. In addition, left area 44 was significantly interconnected with the MCC. Experts in creative writing differed from non-experts by an increased rFC between right caudate and right IPS and a reduced interhemispheric rFC between BA 44 and IPS and caudate.

Across all participants, behavioral creativity scores were predominantly inversely correlated to interhemispheric and left-intrahemispheric rFC. Only rFC between the left IPS and the right caudate correlated positively with the creativity rating of the text. The same pattern was observed in experts, since left-hemispheric and interhemispheric rFC was negatively associated with creativity, apart from positive correlations with the rFC of the right caudate and left IPS and of the right area 44 and the right posterior STS.

### Decreased left- and interhemispheric rFC in experts of creative writing

rFC in experts was characterized by a reduced left- and interhemipheric integration. These findings corroborate previous results of EEG coherence measures that demonstrated less cooperation of brain areas related to creative thinking (Jausovec and Jausovec, 2000). Here verbal creativity scores were negatively associated with right hemispheric and interhemispheric connectivity between cortical areas (Jausovec and Jausovec, 2000) supporting previous suggestions (Petsche, 1996) that it is the functional relations between brain regions, rather than the localized power measures that prove to be better indicators of individual differences. In a recent review, Jung et al. (2013) described creative cognition as characterized by “blind variation” (idea generation) and “selective retention” (convergent thinking) processes (Simonton, 2013). Within this framework, the default mode network (Raichle et al., 2001) could serve as a system operating disinhibitory mental simulation processes, whereas specific associated cognitive control networks based on excitatory processes would initiate selection processes and refine ideas (Jung et al., 2013). Our data fit to this model insofar as it provided evidence for a reduced left- and interhemispheric integration of language areas (especially left area 44) that may lead to a more autonomous and less constraining functioning of separate elements of the network. Previous rFC data (Takeuchi et al., 2012; Wei et al., 2014) have stressed the involvement of the default mode network in creative cognition.

### The role of the left caudate in verbal creativity tasks

Concerning the role of the basal ganglia in creative cognition, Abraham et al. (2012a) found better scores in a creativity task that demands to overcome the constraining influences of salient examples in a group of patients with basal ganglia lesions. The fact that patients with lesions of the basal ganglia may perform better in problem solving tasks requiring to ignore pieces of information fits well with the literature on inhibitory control operations that are considered a central function of the basal ganglia. Basal ganglia lesions thus result in poor inhibitory control, inattention and increased distractibility (Aron et al., 2003), which is advantageous in overcoming knowledge constraints. In the present study, verbal creativity of experts was correlated to reduced FC of the left caudate with left temporal pole, what is in agreement with an enhanced verbal creativity going along with a decreased inhibition. The left temporal pole is considered a semantic “hub” in the brain (Patterson et al., 2007) and has been found involved in verbal (Abraham et al., 2012b) and non-verbal (Ellamil et al., 2012) creativity tasks, as well as in figurative language comprehension, such as metaphors (Schmidt and Seger, 2009; Mihov et al., 2010). Disinhibition of the left temporal pole may thus contribute to excellent verbal skills in experts of creative writing.

### Increased rFC between intraparietal sulcus and right caudate in experts

Remarkably, in our study experts showed decreased cortico-cortical left and interhemispheric connectivity but increased right-hemispheric interactions of the caudate with the intraparietal sulcus. Further, rFC between right caudate and left IPS were positively correlated with creativity measures. The IPS is involved in verbal short-term memory and functions as an attentional modulator of distant neural networks which themselves are specialized in processing language representations (Majerus et al., 2006). Bilateral caudate activation in turn has been observed during an untrained working memory task (Moore et al., 2013) as well as during long-term working memory training (Kühn et al., 2013) and seems to mediate changes in underlying working memory ability. Increased rFC between the right caudate and bilateral IPS in experts of creative writing may thus be connected to their special expertise and practice with handling verbal information and not be an expression for verbal creativity *per se*.

Although rFC between two regions need not be based on direct anatomical connectivity, the following white-matter fibers interconnecting our seeds might be relevant here: all the interhemispheric connections (commissural fibers) are passing the corpus callosum. These connections have been identified between the inferior parietal sulci, the caudate nuclei, and the areas 44. As for *intra*hemispheric association fibers, the superior longitudinal fascicle III (Schmahmann et al., 2007) might be most relevant. This structure connects the IPS with ipsilateral area 44 on both hemispheres. Furthermore, the inferior longitudinal fascicle might be the relevant association fiber connecting the left hippocampus to the left temporal pole. Parts of the cingular bundle (Schmahmann et al., 2007) might interconnect left area 44 with the MCC. In addition, the right caudate and right IPS, whose functional interconnection was changed in the expert group, might be connected by the fronto-occipital fasciculus (Schmahmann et al., 2007).

### Limitations

There are several limitations for the approach used in this study. One is that we selected baseline periods of a rest/activation blocked design study instead of measuring a single continuous resting-state period (cf. Fair et al., 2007). Since the number of scans used for our analysis is lower than in comparable resting-state analyses, our approach may have reduced statistical power, potentially leading us to miss relevant but smaller expertise effects. Therefore, further investigations of the effects of verbal creativity on brain networks using longer, continuous resting-state time series would be desirable.

In addition, it can not be excluded that there may be a carry-over of the BOLD response from the previous block. Since we did not investigate ROIs of the default mode networks, as did others (Takeuchi et al., 2012; Wei et al., 2014), we are not able to comment on their findings of increased medial prefrontal lobe rFC for creativity. Therefore, future studies might take a more exploratory approach to be able to encompass a wider set of regions associated with verbal creativity.

Finally, it has to be kept in mind that verbal creativity scores and practice in professional writing were associated in our participants. This association is inherent in the expertise approach chosen here to study neural correlates of verbal creativity. Therefore, our approach does not allow for disentangling influences of practice and innate predisposition (i.e., talent).

## Conclusion

We here reported on the first comparison of rFC in an expert group in creative writing relative to a closely matched control group. Experts exhibited a reduced interhemispheric rFC and negative correlations of creativity scores with rFC between left caudate and left temporal pole which may indicate less inhibition and more autonomous functioning of language areas. On the other hand, rFC between the right caudate and IPS may reflect long-term experience with verbal information processing. Future studies might use modulation procedures to investigate changes in cortical interaction for different phases of creative verbal processes. In addition, a closer focus on basal ganglia cortical interaction, for instance in patients with basal ganglia lesions, could be an interesting direction for new research.

## Conflict of interest statement

The authors declare that the research was conducted in the absence of any commercial or financial relationships that could be construed as a potential conflict of interest.
